# The optimal neoadjuvant treatment strategy for HR+/HER2 + breast cancer: a network meta-analysis

**DOI:** 10.1038/s41598-024-84039-2

**Published:** 2025-01-03

**Authors:** Shiwei Liu, Miao Yu, Exian Mou, Meihua Wang, Shuanghua Liu, Li Xia, Hui Li, Hao Tang, Yajing Feng, Xin Yu, Kun Mi, Hao Wang

**Affiliations:** 1 Department of Breast, Sichuan Clinical Research Center for Cancer, Sichuan Cancer Hospital & Institute, Sichuan Cancer Center, Affiliated Cancer Hospital of University of Electronic Science and Technology of China, Chengdu, China; 2 Radiation Oncology Key Laboratory of Sichuan Province, Sichuan Clinical Research Center for Cancer, Sichuan Cancer Hospital & Institute, Sichuan Cancer Center, Affiliated Cancer Hospital of University of Electronic Science and Technology of China, Chengdu, China; 3 School of Medical and Life Sciences, Chengdu University of Traditional Chinese Medicine, Chengdu, China; 4 School of Medicine, University of Electronic Science and Technology of China, Chengdu, China; 5 Shanghai Roche Pharmaceuticals Ltd, Shanghai, China

**Keywords:** Breast cancer, HER2 positive, HR positive, Neoadjuvant therapy, Targeted therapy, Network meta-analysis, Breast cancer, Targeted therapies

## Abstract

The efficacy of neoadjuvant therapy varies significantly with hormone receptor (HR) status for patients with human epidermal growth factor receptor 2 (HER2) positive breast cancer (BC). Despite extensive research on HER2 + BC, the optimal neoadjuvant strategy for HR+/HER2 + BC remains inconclusive. This study aimed to identify the optimal neoadjuvant regimen for HR+/HER2 + BC treatment. We conducted a systematic search for trials comparing neoadjuvant regimens for HR+/HER2 + BC and a network meta-analysis. Odds ratios for pathological complete response (pCR) and hazard ratios for event-free survival (EFS) were calculated. Treatment regimens were ranked using the surface under the cumulative ranking curve. 20 trials with 2809 patients were included. In pCR analysis, three neoadjuvant regimens sequentially ranked at the top, namely those comprising T-DM1, pertuzumab with trastuzumab, and tyrosine kinase inhibitor with trastuzumab, demonstrating significantly higher pCR rates than monotherapies. In EFS analysis, pertuzumab with trastuzumab ranked the first while T-DM1 containing regimen ranked the last. Anthracycline-free regimens showed a marginally higher pCR rate than anthracycline-containing regimens, while carboplatin-containing regimens demonstrated a numerically higher pCR rate than carboplatin-free regimens. Significant heterogeneity was observed in endocrine therapy analysis, which may be caused by different strategies for incorporating endocrine therapy. In conclusion, trastuzumab plus pertuzumab stands out as the optimal neoadjuvant HER2-targeting regimen for HR+/HER2 + BC Furthermore, anthracycline-free carboplatin-containing chemotherapy emerges as a promising combination treatment. Further investigation is required to clarify the role of endocrine therapy in HR+/HER2 + BC to guide its clinical application.

**Supplementary Information:**

The online version contains supplementary material available at 10.1038/s41598-024-84039-2.

## Introduction

Neoadjuvant treatment is highly recommended for patients with human epidermal growth factor receptor 2-positive (HER2+) breast cancer (BC), given its well-established benefits in improving surgery outcome and survival^1,2^. Pathological complete response (pCR) is a standard endpoint for measuring the short-term outcome of neoadjuvant treatment, while the long-term endpoints are often survival outcomes^3^. Approximately 50–70% of HER2 + BC cases also express hormone receptors (HR), which have a complex crosstalk with HER2^4,5^. The HR-positive (HR+)/HER2 + population faces higher treatment resistance and a less favorable prognosis^6^. Achieving pCR in neoadjuvant therapy is more challenging in the HER2+/HR + population when compared with the HER2+/HR-negative (HR-) population^7^. This highlights an unmet clinical need to determine the optimal neoadjuvant strategy for HR+/HER2 + BC. Commonly used regimens and drugs are summarized in Supplementary Table S1.

Targeted therapy for HER2 + BC has advanced significantly over the past decades. Trastuzumab combined with pertuzumab is the current standard of care for primary HER2 + BC in the neoadjuvant setting^1,2^. Besides, novel HER2-targeting therapies showed promising results in the neoadjuvant setting, such as antibody-drug conjugate trastuzumab emtansine (T-DM1)^8,9^ or trastuzumab deruxtecan (T-DXd)^10^ and tyrosine kinase inhibitor (TKI) neratinib^11^ or pyrotinib^12^. These trials confirmed that the HR + population achieved lower pCR rates than the HR- population. Notably, no head-to-head comparison of all regimens has been conducted in the HR+/HER2 + population, raising the question of whether the HR + population might benefit from different HER2-targeting regimens compared to the overall HER2 + population.

Chemotherapy exhibits reduced benefits in the HR + BC population compared to HR- BC^13^. Therefore, there is a growing trend to minimize chemotherapy to balance risks and benefits^14^. Conventionally, anthracyclines were frequently used in chemotherapy regimens. However, recent studies have shown that the cardiotoxicity of anthracyclines may be superimposed with antibodies^15^, and omitting the use of anthracyclines does not influence pCR or survival outcomes in patients with HER2 + BC^16^. The TRYPHAENA trial added carboplatin to anthracycline-free neoadjuvant chemotherapy, which yielded similar pCR and event-free survival (EFS) between both groups^17^, establishing the role of carboplatin in treating HER2 + BC. Therefore, there are clinical considerations regarding whether anthracyclines could be omitted and whether carboplatin should be added to neoadjuvant chemotherapy regimens for HR+/HER2 + BC treatment.

Due to the complex interaction between the downstream pathways of HR and HER2, endocrine therapy (ET) is recommended for HR+/HER2 + BC treatment^18,19^. Dual pathway blockade has demonstrated considerable efficacy in advanced BC^20–22^. Therefore, neoadjuvant ET has been well studied in the HR+/HER2 + population and yielded positive outcomes^23–26^. Nonetheless, debates persist regarding the clinical applications of neoadjuvant ET, including its optimal timing, potential long-term impact on prognosis, the suitability of pCR as an efficacy endpoint, and its potential to replace chemotherapy.

Previous network meta-analyses have primarily focused on the overall HER2 + BC population, with limited exploration of the HR+/HER2 + subgroup.^16,27–30^, While a few studies have evaluated pCR rates across regimens for HR+/HER2 + BC, these analyses often lacked comprehensive consideration of long-term outcomes^16,30,31^.

Leveraging newly released data from recent trials, such as WGS-TP-II^32^ and PHEDRA^12^, this study aims to demonstrate the optimal HER2-targeting regimen for HR+/HER2 + BC by integrating both short-term and long-term outcomes. Additionally, we aimed to explore the roles of carboplatin, anthracycline, and ET in the neoadjuvant period, providing updated insights into the management of this challenging subgroup.

## Materials and methods

### Search strategy and selection criteria

This study is a systematic review with network meta-analysis. Our review protocol was registered on the PROSPERO website (CRD42023385644), and this systematic review is reported following the Preferred Reporting Items for Systematic Reviews and Meta-Analyses (PRISMA) guidelines and its extension for network meta-analysis^33–35^.

Literature was retrieved from Medline, EMBASE, Cochrane Library, and Web of Science with no restrictions on publication year or language. Detailed strategies used in each database are provided in Supplementary Table S2–S5. Search terms were built based on population, intervention, and outcome of interest. References of formerly published trials and reviews were also checked for missing literature. Unpublished trials and gray literature were not included.

The predefined inclusion and exclusion criteria are as follows: Neoadjuvant clinical trials were eligible if they met all the following criteria: (1) included patients with HR+/HER2 + BC, (2) compared any neoadjuvant regimens that included HER2-targeting therapies, and (3) reported any pCR or EFS (or disease-free survival) outcome. Trials were excluded if they (1) were bioequivalence studies, (2) did not report the outcome of patients with HR+/HER2 + BC, and (3) compared solely on variations in dose or sequence between treatment regimens. The literature retrieved was selected by two reviewers independently.

### Interventions

Interventions were merged according to HER2-targeting therapies of the neoadjuvant regimens, and interventions of interest included six types as follows:

1. T-DM1-based regimens (T-DM1), which include T-DM1 alone, T-DM1 + pertuzumab, or T-DM1 + lapatinib, either with or without chemotherapy or ET.
2. trastuzumab and pertuzumab with chemotherapy (HP + C).
3. trastuzumab and TKI with chemotherapy (H + TKI + C). TKI includes lapatinib, neratinib, and pyrotinib.
4. trastuzumab with chemotherapy (H + C).
5. lapatinib with chemotherapy (L + C).
6. any chemotherapy with no HER2-targeting regimen (C).

### Outcomes

The primary outcome of this study was the number of patients achieving pCR, defined as the absence of residual invasive cancer in the breast and no pathological involvement of axillary lymph nodes (ypT0/Tis ypN0). Other definitions of pCR were also accepted as (1) the absence of residual invasive and in situ cancer in the breast and no pathological involvement of axillary lymph nodes (ypT0 ypN0) or (2) the absence of residual invasive cancer in the breast (ypT0/Tis) since some trials did not report ypT0/Tis ypN0, and sensitivity analysis was conducted to assess the potential bias involved by this. The secondary outcome was EFS, defined as the time from randomization to an event which may include disease progression, discontinuation of the treatment for any reason, or death.

### Data analysis

All data were independently extracted by two reviewers. Data were extracted on summary estimates, including trial characteristics (trial name, register number, starting year, country, and total participants), patient characteristics (age, tumor stage, tumor size, menopausal status, and HR status), intervention details (regimen and total duration), and interested outcomes. For pCR, the number of patients and the pCR definitions in the corresponding trial were extracted. For EFS, three-year or five-year EFS rates, hazard ratios (HRs), and the 95% confidence interval (CI) between treatment arms were extracted.

All studies included were assessed for the risk of bias with the Cochrane risk-of-bias tool (RoB2)^36^ by two reviewers independently. Five domains listed below were checked and reported as low risk, some concern, or high risk for each trial.

1. Bias arising from the randomization process, assessing the generation and concealment of allocation sequence, and the differences between intervention groups.
2. Bias due to deviations from intended interventions, assessing the blinding of participants and personnel, and deviations from the intended interventions.
3. Bias due to missing outcome data, assessing the availability of outcome data.
4. Bias in the measurement of the outcome, assessing the appropriateness and blinding of outcome assessment.
5. Bias in the selection of the reported result.

All statistical analyses were conducted based on the HR+/HER2 + BC population. For each comparison of regimens reported in more than one trial, we performed a direct meta-analysis with odds ratios (ORs) of pCR for pooling effect sizes. A common effect model was used, and ORs were reported with 95% CIs, where a random effects model was also conducted for sensitivity analysis. All tests were two-sided and statistical significance was set at *p* < 0·05. Cochran’s Q test was performed to assess the statistical heterogeneity. For comparison groups with *I*^2^ statistic > 25% or p-value of the Q test < 0·1, sensitivity analysis was conducted to identify the source of heterogeneity by deleting each trial. Peter’s test was conducted to assess publication bias in each direct comparison, and results were presented by funnel plots^37^.

For indirect comparisons, we conducted a network meta-analysis with the common effect model in a Bayesian framework. Four Markov chains were run for 50,000 simulation iterations after 10,000 tuning iterations. ORs with 95% CIs for pCR analysis and HRs with 95% CIs for EFS analysis were reported. A node-splitting analysis was performed to determine the inconsistency between direct and indirect comparisons^38^, and a comparison-adjusted funnel plot was constructed to evaluate the small-study effects across the network^39^. Sensitivity analysis was performed by reevaluating the results while excluding trials with a high risk of overall bias, using other TKIs than lapatinib, or reporting pCR outcomes in different definitions. The ranking probability was evaluated for each treatment regimen and summarized with the surface under the cumulative ranking curve (SUCRA). A clustered SUCRA ranking plot considering both pCR and EFS was also presented^40^.

To further analyze the efficacy of chemotherapy and ET regimens, direct comparisons were performed between anthracycline-containing and non-anthracycline therapy, carboplatin-containing and non-carboplatin therapy, as well as regimens with or without ET. Heterogeneity was assessed with Cochran’s Q test, and subgroup analysis was performed to identify its source.

All statistical analyses were conducted in R version 4.3.1. Plots in risk of bias analysis were made with the ‘robvis’ package. Direct meta-analysis was performed with ‘meta’ package. Bayesian network meta-analysis was performed with ‘gemtc’ and ‘rjags’ package.

## Results

### Literature retrieval and study characteristics

**Fig. 1:**
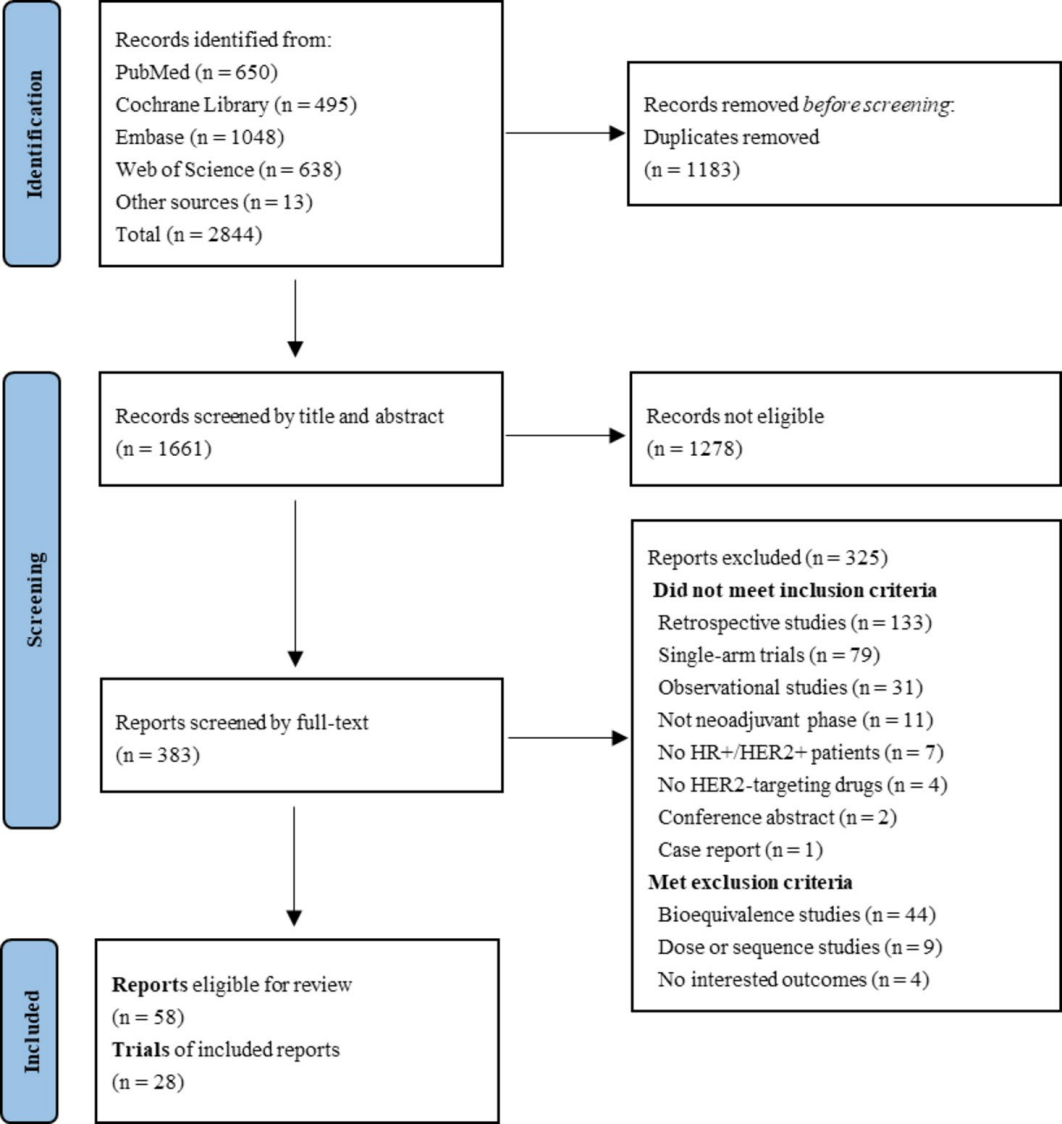
PRISMA flow chart of literature retrieval and selecting.

2844 articles were retrieved from four databases, of which 58 articles^8,9,11,12,17,25,26,32,41–90^, including 28 trials, were eligible and were extracted for accessible data (Fig. 1; Table 1). 20 trials with 2809 patients with HR+/HER2 + BC were included in the primary pCR analysis comparing HER2-targeting regimens. Additionally, five trials with 877 patients were assessed for ET efficacy, six trials with 818 patients for carboplatin, and four trials with 482 patients for anthracycline.

The duration of neoadjuvant treatment ranged from 12 to 30 weeks, due to differences in cycle numbers and length. Among all HER2 + cases included, 63·0% (4347/6904) were HR+. All trials reported the number of patients with pCR in the HR + subgroup, while six of them provided EFS results for the HR + subgroup.

The risk of bias assessment identified one trial with a high overall risk of bias due to the missing randomization process. Most trials (21/28) had a low overall risk of bias, while six trials had some concerns about the risk of bias (Supplementary Fig. S1, Supplementary Table S6).

**Table 1:** Patient characteristics of the include trials.

Trial name	Country	Year	Register number	Number of participants	Regimen type	Detailed regimen	Duration (weeks)	Number of patients each arm	Median age	Post-menopausal	Breast cancer stage			T stage					Median tumor size (mm)	HR+	Number of HR + patients achieving pCR	EFS rate in HR + patients	HRs of EFS (95% CI)	Follow-up years
											I	II	III	T0	T1	T2	T3	T4						
Buzdar, 2005	USA	2001	-	42	C	T→F + E + C	24	19	48 (25–75)	-	-	-	-	-	2	13	4	0	-	11	3	-	-	-
					H + C	T + H→F + E + C + H	24	23	52 (29–71)	-	-	-	-	-	2	15	5	1	-	13	8	-	-	-
REMAGUS 02	France	2004	ISRCTN10059974	120	C	E + C→D	24	58	-	-	-	-	-	-	-	27	31	-	-	37	7	-	-	-
					H + C	E + C→D + H	24	62	-	-	-	-	-	-	-	32	30	-	-	34	7	-	-	-
CherLOB	Italy	2006	NCT00429299	121	H + C	T + H→F + E + C + H	26	36	50 (34–65)	19 (52.8%)	0	30	6	-	-	-	-	-	-	21	5	-	-	-
					L + C	T + L→F + E + C + L	26	39	49 (34–68)	24 (61.5%)	0	32	23	-	-	-	-	-	-	24	5	-	-	-
					H + TKI + C	T + H + L→F + E + C + H + L	26	46	49 (26–65)	27 (58.3%)	0	13	9	-	-	-	-	-	-	28	10	-	-	-
NeoSphere	International	2007	NCT00545688	417	H + C	D + H	12	107	50 (32–74)	-	-	-	-	-	-	-	-	-	50 (20–200)	50	10	0.86	References	5
					HP + C	D + H + P	12	107	50 (28–77)	-	-	-	-	-	-	-	-	-	55 (20–150)	50	13	0.87	0.86 (0.27, 2.75)	
						H + P	12	107	49 (22–80)	-	-	-	-	-	-	-	-	-	50 (20–200)	51	3	-	-	-
						D + P	12	96	49 (27–70)	-	-	-	-	-	-	-	-	-	50 (0–180)	46	8	-	-	-
GeparQuinto	Germany	2007	NCT00567554	615	H + C	EC + H→D + H	12	307	50 (25–74)	-	5	159	141	-	251			56	-	170	43	0.88	References	3
					L + C	EC + H→D + L	12	308	50 (21–73)	-	3	141	152	-	253			55	-	171	26	0.905	0.87 (0.52, 1.47)	
LPT109096	USA	2007	NCT00524303	100	H + C	F + E + C + H→T	24	33	54 (21–67)	19 (58%)	-	-	-	-	-	22	8	3	-	13	5	-	-	-
					L + C	F + E + C + L→T	24	34	52 (25–67)	19 (56%)	-	-	-	-	-	12	11	8	-	13	3	-	-	-
					H + TKI + C	F + E + C + H + L→T	24	33	50 (28–66)	16 (48%)	-	-	-	-	-	22	6	5	-	15	11	-	-	-
NSABP B-41	USA	2007	NCT00486668	529	H + C	A + C→T + H	28	181	-	-	-	-	-	-	-	102	79	-	-	122	55	-	-	-
					L + C	A + C→T + L	28	174	-	-	-	-	-	-	-	81	93	-	-	100	42	-	-	-
					H + TKI + C	A + C→T + H + L	28	174	-	-	-	-	-	-	-	88	86	-	-	108	59	-	-	-
Wang, 2017	China	2007	-	549	H + C	T + Cb + H + Endo	16	379	-	225 (59.4%)	-	-	-	-	-	84	244	51	-	187	75	-	-	-
					C	T + Cb + Endo	16	170	-	102 (60.0%)	-	-	-	-	-	36	114	20	-	82	15	-	-	-
CALGB 40,601	USA	2008	NCT00770809	299	H + TKI + C	T + H + L	16	118	48 (24–70)	41 (35%)	0	80	37	-	-	-	-	-	40 (14–220)	68	28	-	-	-
					H + C	T + H	16	120	50 (30–75)	52 (44%)	0	80	38	-	-	-	-	-	40 (12–150)	70	29	-	-	-
					L + C	T + L	16	67	50 (25–74)	27 (42%)	0	47	17	-	-	-	-	-	40 (11–120)	35	10	-	-	-
NeoALTTO	International	2008	NCT00553358	455	L + C	L→T + L	18	154	50 (42–56)	-	-	-	-	-	-	-	-	-	-	80	13	0.73	0.86 (0.44, 1.63)	6
					H + C	H→T + H	18	149	49 (44–57)	-	-	-	-	-	-	-	-	-	-	75	17	0.71	References	
					H + TKI + C	L + H→T + H + L	18	152	50 (43–59)	-	-	-	-	-	-	-	-	-	-	77	32	0.74	0.80 (0.42, 1.52)	
TRIO-US B07	USA	2008	NCT00769470	128	H + C	H→T + Cb + H	21	34	48	-	2	20	12	-	-	-	-	-	55.4 (10–200)	20	8	-	-	-
					L + C	L→T + Cb + L	21	36	51	-	1	28	7	-	-	-	-	-	51.6 (20–140)	18	2	-	-	-
					H + TKI + C	H + L→T + Cb + H + L	21	58	47	-	3	38	17	-	-	-	-	-	41.5 (14–120)	34	14	-	-	-
TRYPHAENA	International	2009	NCT00976989	225	HP + C	F + E + C + H + P→D + H + P	18	73	49 (27–77)	-	-	-	-	-	-	-	-	-	53 (10–220)	39	18	-	-	-
					HP + C	F + E + C→D + H + P	18	75	49 (24–75)	-	-	-	-	-	-	-	-	-	49 (19–120)	35	17	-	-	-
					HP + C	D + Cb + H + P	18	77	50 (30–81)	-	-	-	-	-	-	-	-	-	50 (15–200)	40	20	-	-	-
EORTC 10,054	Europe	2010	NCT00450892	128	L + C	D + L→F + E + C	18	23	*49.9 (27.3–68.5)	-	-	-	-	0	1	11	8	3	-	14	6	-	-	-
					H + C	D + H→F + E + C	18	53	*47.0 (25.3–68.9)	-	-	-	-	0	0	24	19	10	-	27	14	-	-	-
					H + TKI + C	D + L + H→F + E + C	18	52	*49.4 (27.3–70.8)	-	-	-	-	1	0	28	13	9	-	23	11	-	-	-
NSABP FB-7	International	2011	NCT01008150	126	H + C	T + H→A + C + T + H	28	42	50 (33–71)	17 (40%)	0	18	21	-	-	-	-	-	37 (0-180)	27	8	-	-	-
						T + N→A + C + T + N	28	42	56 (29–71)	26 (62%)	0	19	20	-	-	-	-	-	55 (0-100)	29	8	-	-	-
					H + TKI + C	T + H + N→A + C + T + H + N	28	42	50 (31–77)	19 (45%)	0	20	22	-	-	-	-	-	50 (0–90)	23	7	-	-	-
NeoLaTH	Japan	2012	UMIN000007576	213	H + TKI + C	H + L→T + H + L	18	44	56 (33–69)	30 (68%)	-	-	-	-	4	31	9	-	29 (13–62)	0	0	-	-	-
					H + TKI + C	H + L→T + H + L	30	48	56 (36–69)	30 (63%)	-	-	-	-	6	29	13	-	32 (14–70)	0	0	-	-	-
					H + TKI + C	H + L→T + H + L	18	41	52 (32–70)	22 (54%)	-	-	-	-	11	26	4	-	24 (11–73)	41	13	-	-	-
					H + TKI + C	H + L + Endo→T + H + L + Endo	18	40	53.5 (26–66)	20 (50%)	-	-	-	-	8	27	5	-	28 (11–60)	39	13	-	-	-
					H + TKI + C	H + L + Endo→T + H + L + Endo	30	40	49 (28–68)	20 (50%)	-	-	-	-	11	26	3	-	27 (14–61)	40	15	-	-	-
WSG-ADAPT HER2+/HR+	Germany	2012	NCT01779206	375	T-DM1	T-DM1	12	119	-	-	-	-	-	-	60	51	6	2	-	117	48	0.889	References	5
					T-DM1	T-DM1 + Endo	12	127	-	-	-	-	-	-	62	58	7	0	-	123	51	0.853	1.47 (0.70, 3.08)	
						H + Endo	12	129	-	-	-	-	-	-	60	61	6	2	-	119	18	0.846	1.57 (0.75, 3.30)	
I-SPY2 T-DM1	USA	2013	NCT01042379	263	T-DM1	T-DM1 + P	12	52	48 (33–72)	-	-	-	-	-	-	-	-	-	-	35	18	0.884	0.98 (0.42, 2.28)	5
					HP + C	T + H + P	12	45	47 (29–70)	-	-	-	-	-	-	-	-	-	-	29	14	0.957	0.42 (0.04, 4.50)	
					H + C	T + H	12	31	50 (29–71)	-	-	-	-	-	-	-	-	-	-	19	3	0.83	References	
TRAIN-2	Netherlands	2013	NCT01996267	438	HP + C	F + E + C + H + P →T + Cb + H + P	27	219	49 (43–55)	99 (45%)	-	-	-	148			71		-	125	64	-	-	-
					HP + C	T + Cb + H + P	27	219	48 (43–56)	96 (44%)	-	-	-	154			65		-	116	64	-	-	-
PREDIX HER2	Sweden	2014	NCT02568839	198	HP + C	D + H + P	18	99	51 (26–73)	48 (49.5%)	-	-	-	-	14	63	17	-	-	66	24	-	-	-
					T-DM1	T-DM1	18	99	53 (28–74)	50 (53.2%)	-	-	-	-	20	61	17	-	-	59	21	-	-	-
Neopeaks	Japan	2014	UMIN000014649	204	HP + C	D + Cb + H + P	18	51	53 (28–70)	23 (45.1%)	-	-	-	-	11	37	3	-	27.0 (11–58)	30	13	-	-	-
					T-DM1	D + Cb + H + P →T-DM1 + P + Endo	24	52	53 (29–69)	23 (44.2%)	-	-	-	-	13	35	4	-	25.5 (12–56)	29	20	-	-	-
					T-DM1	T-DM1 + P + Endo	18	80	51.5 (25–70)	39 (48.8%)	-	-	-	-	14	58	8	-	27.0 (11–70)	44	24	-	-	-
					T-DM1	T-DM1 + P + Endo→F + E + C	24	21	53 (40–67)	9 (42.9%)	-	-	-	-	6	14	1	-	27.0 (12–51)	15	6	-	-	-
TEAL	USA	2014	NCT02073487	30	HP + C	H + P →T + H + P	18	16	*57.2 (39.6–74.9)	-	-	7	9	-	-	-	-	-	-	8	2	-	-	-
					T-DM1	T-DM1 + L→T + T-DM1 + L	18	14	*53.1 (27.8–69.7)	-	-	8	6	-	-	-	-	-	-	8	6	-	-	-
KRISTINE	International	2014	NCT02131064	444	T-DM1	T-DM1 + P	18	223	50 (42–57)	-	-	-	-	-	-	-	-	-	-	131	46	0.922	1.44 (0.54, 3.88)	3
					HP + C	D + Cb + H + P	18	221	49 (41–57)	-	-	-	-	-	-	-	-	-	-	128	56	0.917	References	
PEONY	China	2016	NCT02586025	329	HP + C	D + H + P	12	219	49 (24–72)	87 (39.7%)	-	-	-	-	-	155	45	19	-	114	39	-	-	-
					H + C	D + H	12	110	49 (27–70)	45 (40.9%)	-	-	-	-	-	71	29	10	-	56	14	-	-	-
WSG-TP-II	Germany	2017	NCT03272477	207		H + P + Endo	12	100	52 (26–83)	49 (49%)	-	-	-	-	37	57	6	-	-	100	23	-	-	-
					HP + C	T + H + P	12	107	54 (25–79)	63 (59%)	-	-	-	-	49	56	2	-	-	107	57	-	-	-
Dena, 2021	Iran	2017	IRCT2017100136491N1	52	H + C	A + C→T + H	16	27	*45	-	-	-	-	-	7	6	4	10	*31.3	12	7	-	-	-
					H + C	A + C→T + H→Cb + G	22	25	*41	-	-	-	-	-	4	9	5	7	*41.2	11	5	-	-	-
PHERGain	International	2017	NCT03161353	356	HP + C	D + Cb + H + P	12	71	51 (42–58)	34 (48%)	-	-	-	-	9	50	12	-	-	44	21	-	-	-
						H + P + Endo	24	227	50 (45–59)	139 (49%)	-	-	-	-	24	219	42	-	-	157	55	-	-	-
					HP + C	H + P + Endo→D + Cb	24	58			-	-	-	-				-	-	35	7	-	-	-
neoCARH	China	2017	NCT03140553	135	HP + C	E + C→T + H	24	67	50 (25–68)	-	-	-	-	-	3	44	17	3	-	34	9	-	-	-
					HP + C	T + Cb + H	18	68	50.5 (23–68)	-	-	-	-	-	3	43	17	5	-	34	16	-	-	-
PHEDRA	China	2018	NCT03588091	355	H + TKI + C	D + H + Pyr	12	178	50 (43–55)	-	0	128	50	-	-	-	-	-	-	97	29	-	-	-
					H + C	D + H	12	177	50 (44–55)	-	0	125	52	-	-	-	-	-	-	98	12	-	-	-

In detailed regimen, abbreviations are used as follows: T: paclitaxel; D: docetaxel; F: 5’-fluorouracil; E: epirubicin; C: cyclophosphamide; H: trastuzumab; P: pertuzumab; L: lapatinib; N: neratinib; Pyr: Pyrotinib; Cb: carboplatin; A: Doxorubicin; Endo: endocrine therapy; G: gemcitabine; T-DM1: trastuzumab emtansine. Plus(+) stands for simultaneous regimen and arrow(→) stands for sequential regimen. *Average instead of median. -: Not reported.

### Pairwise meta-analysis

**Fig. 2:**
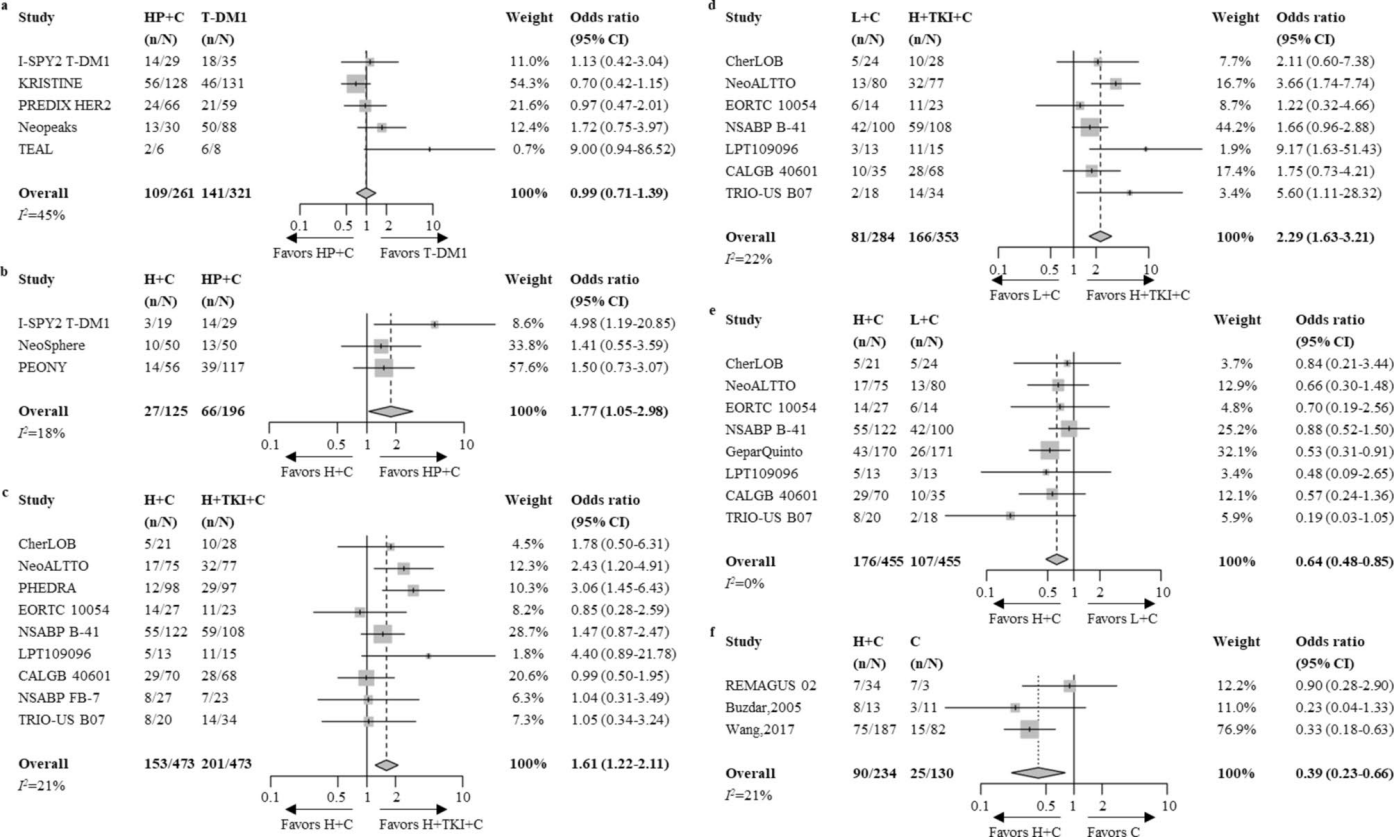
Forest plots of direct comparisons for pCR. (**a**) T-DM1 vs. HP + C; (**b**) HP + C vs. H + C; (**c**) H + TKI + C vs. H + C; (**d**) H + TKI + C vs. L + C; (**e**) L + C vs. H + C; (**f**) C vs. H + C. T-DM1 = trastuzumab emtansine based regimens; HP + C = trastuzumab and pertuzumab with chemotherapy; H + TKI + C = trastuzumab and TKI with chemotherapy; H + C = trastuzumab with chemotherapy; L + C = lapatinib with chemotherapy; C = chemotherapy with no HER2-targeting regimen. The squares and the lines crossing the square stand for OR and its 95%CI. The diamonds stand for the estimated pooled OR and its 95% CI. Heterogeneity was tested with Cochran’s Q test, and all statistical tests were two-sided.

From trials that reported pCR in the HR+/HER2 + population, six direct comparisons were made: (1) T-DM1 vs. HP + C (2) HP + C vs. H + C (3) H + TKI + C vs. H + C (4) H + TKI + C vs. L + C (5) L + C vs. H + C (6) C vs. H + C. HP + C demonstrated a significantly higher pCR rate compared to H + C, with an OR (95% CI) of 1·77 (1·05–2·98). H + TKI + C achieved a higher pCR rate than both H + C and L + C with ORs (95% CI) of 1·61 (1·22–2·11) and 2·29 (1·63–3·21), respectively. Both L + C and C were associated with significantly lower pCR than H + C, with ORs of 0·64 (95% CI 0·48–0·85) and 0·39 (95% CI 0·23–0·66), respectively (Fig. 2). Sensitivity analyses with random effects models all resulted similarly (Supplementary Fig. S2, Supplementary Table S7).

When comparing T-DM1 vs. HP + C, a high *I*^2^ statistic was observed (*I*^*2*^ = 45%, *p* = 0·12) after the heterogeneity test. Sensitivity analysis showed that the lowest *I*^2^ statistic (*I*^*2*^ = 15%, Supplementary Fig. S3) was achieved when the TEAL trial was excluded, with no impact on the outcome of this comparison (OR 0·93, 95%CI 0·66–1·31). No evidence of publication bias was observed after Peter’s test, and all funnel plots were approximately symmetrical (Supplementary Fig. S4).

### Network meta-analysis

**Fig. 3:**
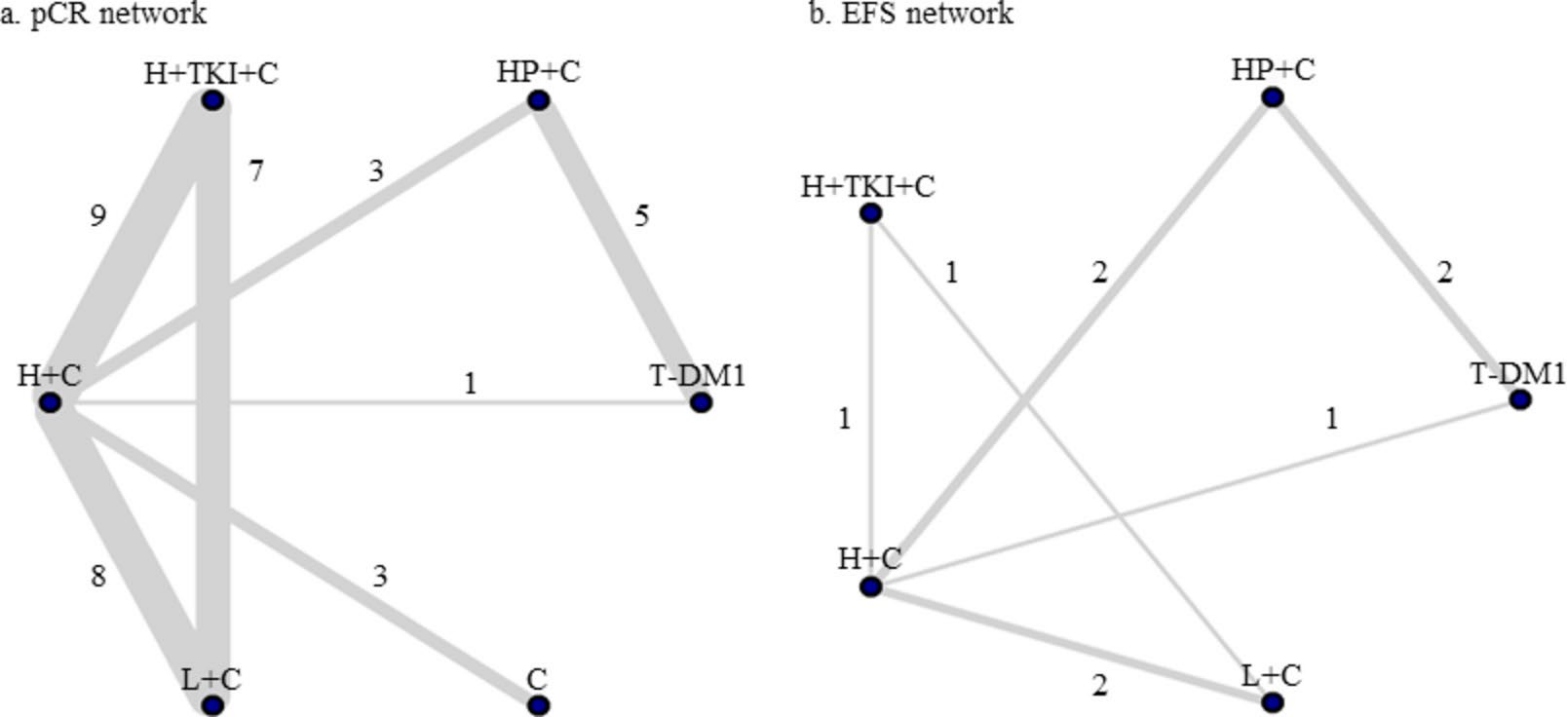
Network plots for pathological complete response (pCR) and event free survival (EFS). (**a**) pCR, and (**b**) EFS. Each line represents a direct comparison, and its width is weighted by the number of trials reporting this comparison, which is presented next to it. T-DM1 = trastuzumab emtansine based regimens; HP + C = trastuzumab and pertuzumab with chemotherapy; H + TKI + C = trastuzumab and TKI with chemotherapy; H + C = trastuzumab with chemotherapy; L + C = lapatinib with chemotherapy; C = chemotherapy with no HER2-targeting regimen.

The network comparing pCR included 20 trials with 15 comparisons, and the network comparing EFS included five trials with ten comparisons (Fig. 3). ORs of pCR and HRs of EFS were listed in Table 2 with their 95% CI. We observed statistically significant differences in 11 comparisons for pCR and no statistically significant difference for EFS across different treatment arms.

The node-splitting model was used for the consistency test, and no significant inconsistency was found (Supplementary Fig. S5). The adjusted funnel plot was symmetrical, indicating no significant small-study effect (Supplementary Fig. S6). Sensitivity analysis was performed by excluding trials with a high risk of overall bias, using other TKIs instead of lapatinib, or reporting pCR in different definitions, and most results remained stable (Supplementary Table S8–S10).

**Table 2:**
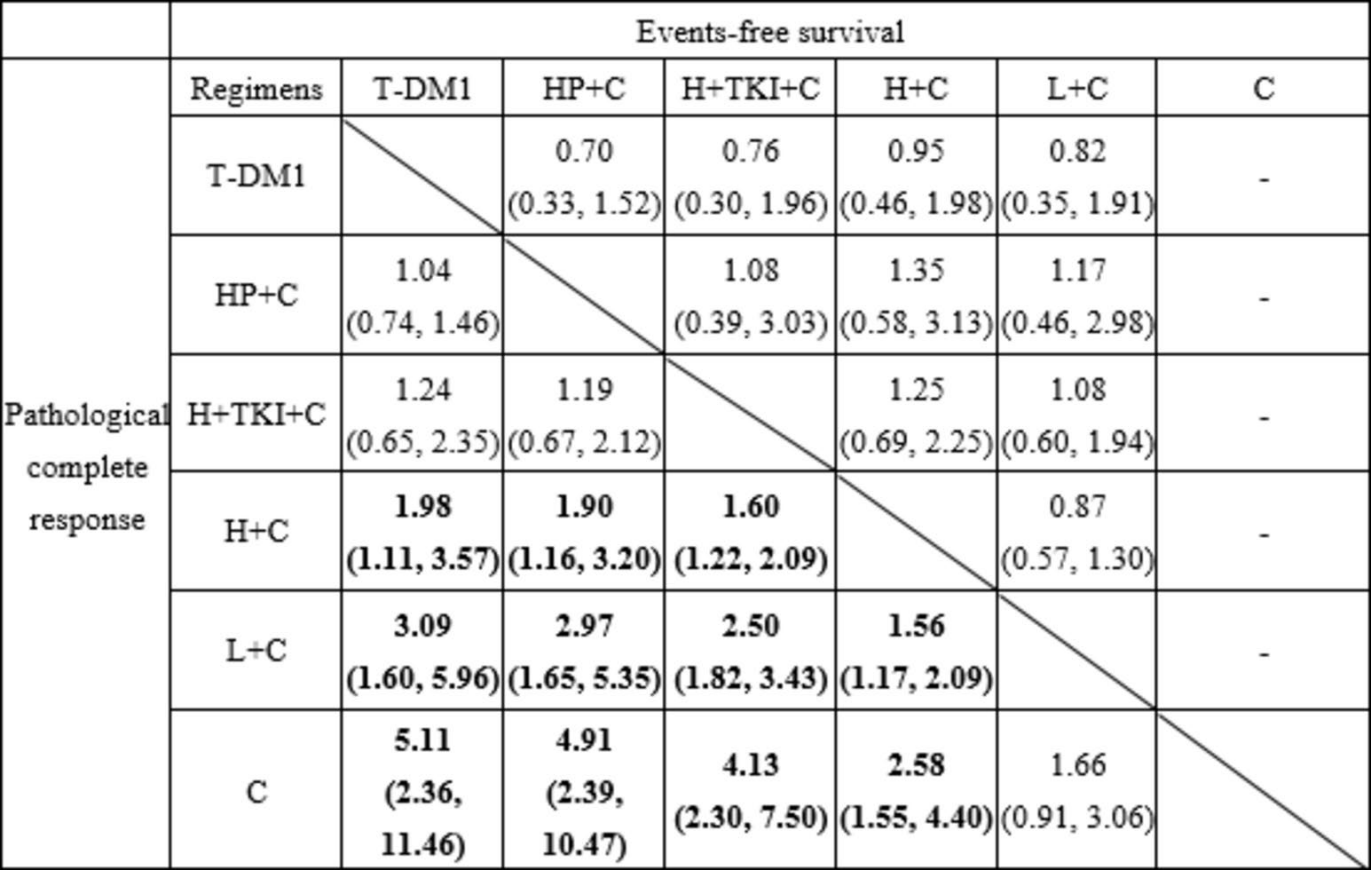
League table of odds ratio for pCR and hazard ratio for EFS.

Odds ratios with 95% confidence intervals comparing pathological complete response (below diagonal line) and hazard ratios with 95% confidence intervals comparing events-free survival (above diagonal line). Results were compared from top to left with significant difference in bold font. T-DM1 = trastuzumab emtansine based regimens; HP + C = trastuzumab and pertuzumab with chemotherapy; H + TKI + C = trastuzumab and TKI with chemotherapy; H + C = trastuzumab with chemotherapy; L + C = lapatinib with chemotherapy; C = chemotherapy with no HER2-targeting regimen.

**Fig. 4:**
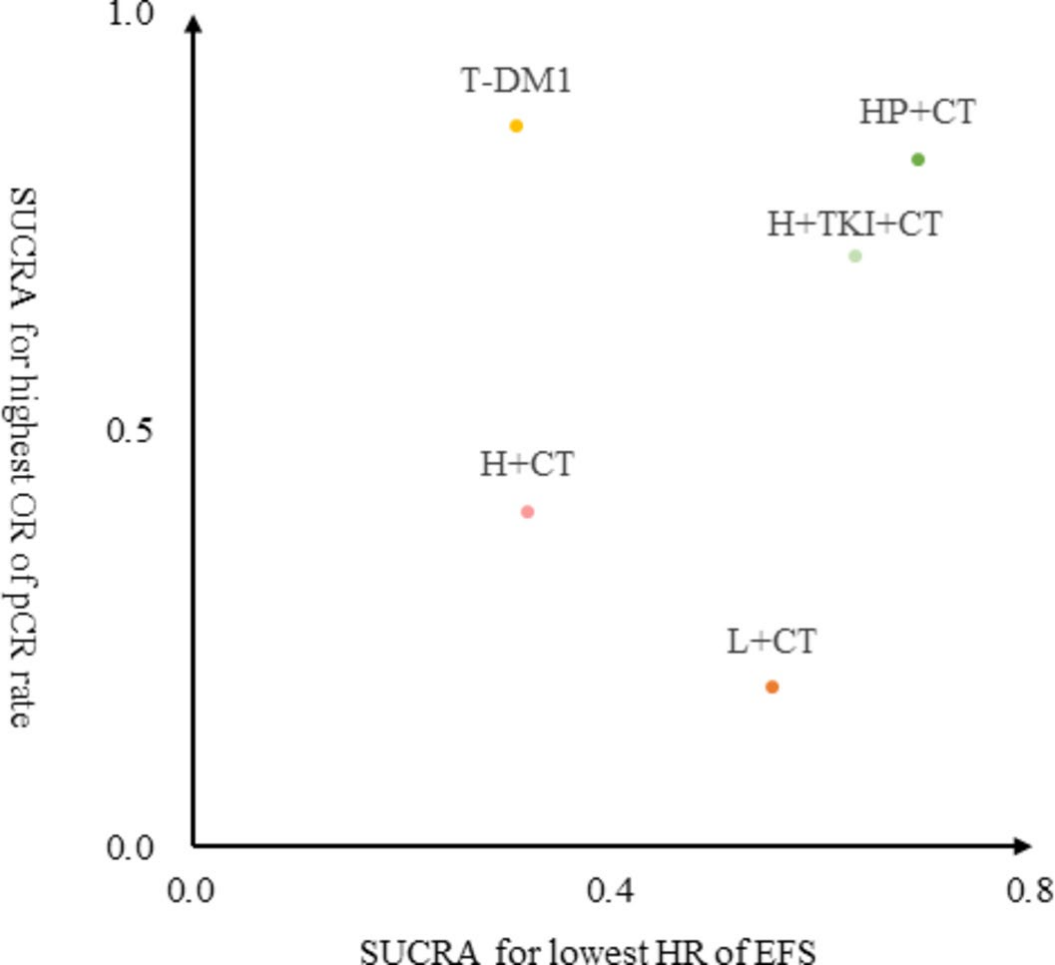
Two-dimensional ranking plot of SUCRA value for highest probability of pCR rate and lowest risk of EFS events. T-DM1 = trastuzumab emtansine based regimens; HP + C = trastuzumab and pertuzumab with chemotherapy; H + TKI + C = trastuzumab and TKI with chemotherapy; H + C = trastuzumab with chemotherapy; L + C = lapatinib with chemotherapy; C = chemotherapy with no HER2-targeting regimen.

### Ranking of regimens

SUCRA values were calculated and indicated that T-DM1 (SUCRA 0.86) had the highest probability of achieving pCR. HP + C (SUCRA 0.82) closely followed in second place, and H + TKI + C (SUCRA 0.71) ranked third. However, when considering EFS, HP + C (SUCRA 0.69) emerged as the top regimen, followed by H + TKI + C (SUCRA 0.63).

A clustered ranking plot was generated by combining the values of SUCRA from both pCR (on the y-axis) and EFS (on the x-axis) (Fig. 4). HP + C emerged as the optimal choice (top right) considering both short-term clinical response and long-term survival outcome. Detailed ranking probabilities are shown in Supplementary Fig. S7.

### Chemotherapy and endocrine therapy analysis

Pairwise meta-analyses were performed to further analyze the efficacy of different chemotherapy and ET with the same HER2-targeting regimen. We conducted three direct meta-analyses comparing pCR rates separately between treatments with or without anthracycline (OR 0·74, 95% CI 0·51–1·07), carboplatin (OR 1·27, 95% CI 0·95–1·96), and ET (OR 0·69, 95% CI 0·52–0·92) (Supplementary Fig. S8 A–C). When comparing ET, significant heterogeneity was observed (*I*^*2*^ = 81%, *p* < 0·01). Therefore, we conducted a subgroup analysis, which indicated that the subgroup where chemotherapy was replaced with ET still presented heterogeneity (*I*^*2*^ = 65%, *p* = 0·09) (Supplementary Fig. S8 D). This heterogeneity could be attributed to variations in neoadjuvant treatment duration. For example, the ET group in the PHERGain trial received 24 weeks of neoadjuvant therapy, while the chemotherapy group in the PHERGain trial and both groups in the WSG-TP-II trial received 12 weeks. Thus, the random effects model was used to compare results with the common effect model (Supplementary Fig. S8 E). For trials incorporating ET alongside other treatments, higher pCR was observed with no statistical significance in the ET group (OR 1·18, 95% CI 0·80–1·73). However, for trials in which chemotherapy was replaced with ET, the pooled result showed a significantly lower pCR rate in ET in both common effect model (OR 0·34, 95% CI 0·22–0·53) and random effects model (OR 0·35, 95% CI 0·16–0·75).

## Discussion

Our findings suggest that the HP + C regimen is the optimal neoadjuvant strategy for HR+/HER2 + BC when considering both pCR and EFS. The anthracycline-free regimen achieved a numerically higher pCR than the anthracycline-containing regimen, and the inclusion of carboplatin also increased the possibility of achieving pCR, although not reaching statistical significance. The efficacy of neoadjuvant ET remains inconclusive.

Both direct and indirect comparisons indicate that the T-DM1-based or dual HER2-targeting regimens are better than single HER2-targeting regimens in improving pCR rates. Though no significant differences were observed in pCR and EFS when comparing T-DM1, HP + C, and H + TKI + C regimens, HP + C had the highest probability of ranking first when considering both pCR and EFS. T-DM1 showed certain advantages in ranking when comparing pCR but ranked lower when comparing EFS. Although the result of the EFS analysis may not be robust enough due to insufficient data, we found that both the KRISTINE^9^ and I-SPY II^8^ trials reported higher risks of EFS events in the T-DM1 group, suggesting T-DM1 may not be the preferred choice for long-term survival benefits. Moreover, results from the KRISTINE^9^ and KAITLIN^91^ trials demonstrated that T-DM1 combined with pertuzumab did not outperform HP + C in either neoadjuvant or adjuvant settings. Thus, further studies are necessary to investigate short-term and long-term benefits of T-DM1 and other antibody-drug conjugates. Based on the existing evidence, the H + TKI + C regimen does not demonstrate significant advantages over the HP + C regimen. Considering that TKIs have a significantly higher incidence of grade 3 or higher adverse events (AEs) in the overall HER2 + population, and there is no evidence suggesting that the risk of AEs is associated with HR status, we could not recommend H + TKI + C over HP + C at present. We noticed that pyrotinib, a new TKI drug, achieved a relatively higher pCR rate^12^. New TKI drugs with improved clinical benefits and safety profiles may hold promise in the future. We conclude that HP + C remains the current optimal neoadjuvant strategy for HR+/HER2 + BC.

The optimal chemotherapy regimen to combine with targeted therapy is controversial. An EBCTCG meta-analysis in the overall BC population demonstrated that adding anthracycline to a taxane regimen yields better outcomes, and higher cumulative doses of anthracycline and taxane are associated with greater benefits^92^. A phase II trial presented at ASCO 2023 showed that albumin-bound paclitaxel combined with trastuzumab and pertuzumab, followed by anthracycline-based regimens, achieved a higher pCR in the HER2+/HR- BC subgroup (81·8%) compared to the HER2+/HR + subgroup (31·6%)^93^. The TRYPHAENA trial reported similar long-term outcomes between groups with or without anthracycline^17^, and the TRAIN-2 trial found that the neoadjuvant treatment of the HR + subgroup favored the non-anthracycline strategy numerically^87^. Our results support the omitting of anthracycline in the neoadjuvant chemotherapy for HR+/HER2 + BC, as we observed higher pCR in anthracycline-free regimens in all included trials, although not statistically significant in the pooled result. Furthermore, the addition of carboplatin to the neoadjuvant chemotherapy regimen appears to increase the likelihood of achieving pCR in this population. Further trials are needed to support this inference since the CI still includes 1.

ET is recommended for HER2+/HR + BC treatment. However, it remains unclear if ET should be initiated in the neoadjuvant period. Our analysis identified significant heterogeneity among trial results, likely due to varied strategies in incorporating ET, including adding ET to standard treatments or replacing chemotherapy with ET. The NSABP B52 trial reported an insignificant improvement in pCR rate for the group adding ET to the HP + C regimen^94^. Our subgroup analysis demonstrated no heterogeneity among the results of studies with ET added to standard treatments, all of which observed higher pCR in the ET group, although the pooled result showed no statistical significance. On the other hand, in trials where neoadjuvant chemotherapy was replaced with ET, relatively low pCR rates were reported. For example, the single-arm trials PAMELA and TBCRC006 reported pCR rates of 18% and 21%, respectively, when ET was combined with trastuzumab and lapatinib without chemotherapy^95,96^. Similar pCR were reported in the PerELISA and PHERGain trials when combining ET with trastuzumab and pertuzumab (21% and 35%, respectively)^23,26^. In our study, only two included trials replaced chemotherapy with ET, both reporting lower pCR in the ET group compared to the chemotherapy group.

For the sole goal of reducing tumor size in neoadjuvant therapy, replacing neoadjuvant chemotherapy with ET may not be optimal, given ET’s lack of a direct inhibitory effect on cancer cell growth. However, neoadjuvant treatment is not limited to local control but is focused on prognosis and guiding postoperative treatment. In this case, even if neoadjuvant ET may not significantly improve the pCR rate, long-term outcomes should be considered when evaluating its benefits. Notably, the three-year follow-up of PHERGain showed that the invasive disease-free survival rate was highest in patients who achieved pCR with ET and without chemotherapy^97^. This indicates that pCR might not be the optimal endpoint for evaluating responses to neoadjuvant ET, and future trials should explore alternative indicators such as preoperative endocrine prognostic index and endocrine sensitive disease rate for their clinical significance.

Patients with HER2+/HR + BC exhibit a lower likelihood of achieving pCR and face poorer prognoses than those with HER2+/HR- BC^6,7^. Our study provided a precise perspective and a focused and detailed analysis on the HR + subgroup of HER2 + BC, addressing an unmet need in this challenging population. Notably, this is the first study to compare EFS across different neoadjuvant regimens specifically for HR+/HER2 + BC, offering insights into both short-term (pCR) and long-term (EFS) outcomes. Furthermore, our work is pioneering in evaluating the role of ET in the neoadjuvant setting for this subgroup.

Some unavoidable limitations should be noted. First, the network of EFS analysis included only five trials, which may limit the robustness of the findings. Second, safety analysis was not conducted due to insufficient data in the HR+/HER2 + population. However, we anticipate a similar safety profile as the overall HER2 + population, which has been extensively studied. Notably, TKI agents are generally associated with more serious AEs than antibodies^16,27,29^. Third, this analysis did not account for variations in TNM staging, the duration of neoadjuvant treatment, or subsequent adjuvant therapy across the included trials. These differences could contribute to heterogeneity in patient cohorts, treatments, and endpoint evaluations.

In conclusion, despite the result being insignificant in EFS comparisons, the HP + C regimen emerged as the optimal neoadjuvant treatment for HR+/HER2 + BC when considering both pCR and EFS outcomes. Additionally, anthracycline-free carboplatin-containing chemotherapy may be a viable combination treatment, although the result did not reach statistical significance. Further studies on neoadjuvant ET are essential to explore appropriate endpoints for evaluating its efficacy and to provide deeper insights into its role in the treatment of HR+/HER2 + BC.

## Electronic supplementary material

Below is the link to the electronic supplementary material.

Supplementary Material 1

## Data Availability

All data included in this study will be shared upon request. Please contact the corresponding author at silentocean@uestc.edu.cn.
