# Supplementary material for: The optimal neoadjuvant treatment strategy for HR+/HER2 + breast cancer: a network meta-analysis
Source: Sci Rep. 2025 Jan 3;15:713. doi: 10.1038/s41598-024-84039-2 (PMC11699132; doi:10.1038/s41598-024-84039-2)
Supplement: Supplementary file 1 — Supplementary Material 1 [file 41598_2024_84039_MOESM1_ESM.pdf]

The optimal neoadjuvant treatment strategy for HR+/HER2+ breast cancer: a network meta-analysis

Shiwei Liu, Miao Yu, Exian Mou, Meihua Wang, Shuanghua Liu, Li Xia, Hui Li, Hao Tang, Yajing Feng, Xin Yu, Kun Mi, and Hao Wang

- Fig. S1. Risk of bias assessment result.
- Fig. S2. Sensitivity analysis with random effects model.
- Fig. S3. Sensitivity analysis with omitting each included trial.
- Fig. S4. Funnel plots assessing publication bias for each direct comparisons.
- Fig. S5. Forest plot of consistency test with node-splitting model.
- Fig. S6. Comparison-adjusted funnel plot of pCR network for pCR and EFS.
- Fig. S7. Ranking probability plot for pCR and EFS comparing result.
- Fig. S8. Forest plots for chemotherapy and endocrine therapy analyses.

- Table S1. Drugs used for neoadjuvant treatment of breast cancer.
- Table S2. Search strategy used in MEDLINE.
- Table S3. Search strategy used in the Cochrane Library.
- Table S4. Search strategy used in Embase.
- Table S5. Search strategy used in Web of Science.
- Table S6. Detailed risk of bias assessment results of each trial.
- Table S7. summarized results of sensitivity analyses of direct comparisons.
- Table S8. League table of odds ratio for pCR in sensitivity analysis removing trials with high risk of overall bias.
- Table S9. League table of odds ratio for pCR in sensitivity analysis removing trials using other TKIs instead of lapatinib.
- Table S10. League table of odds ratio for pCR in sensitivity analysis removing trials reporting pCR outcomes in different definitions.

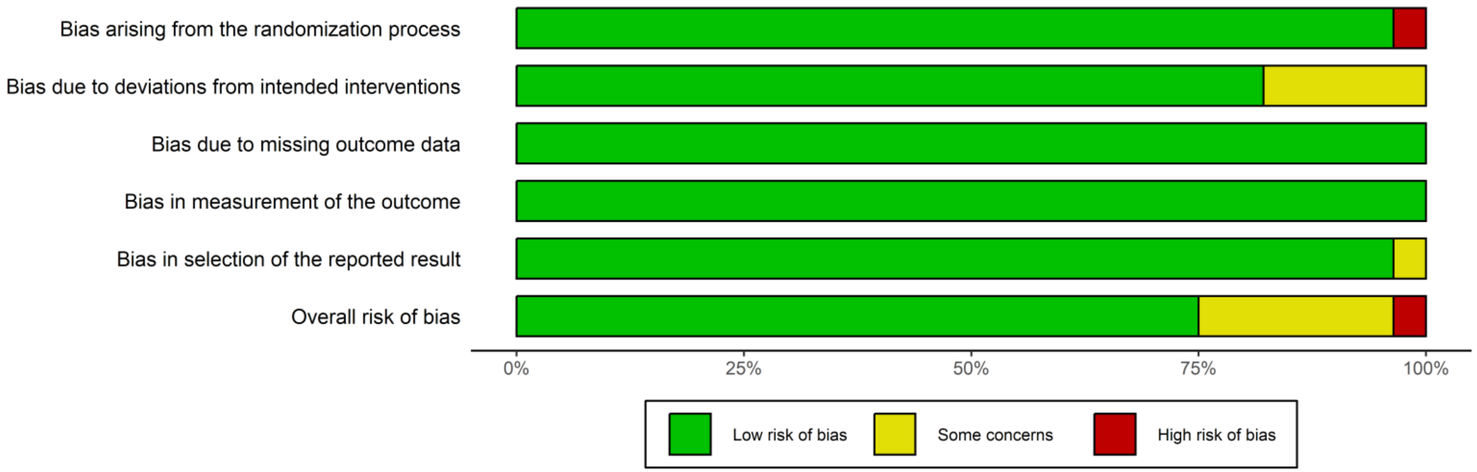

Fig. S1 Risk of bias assessment result

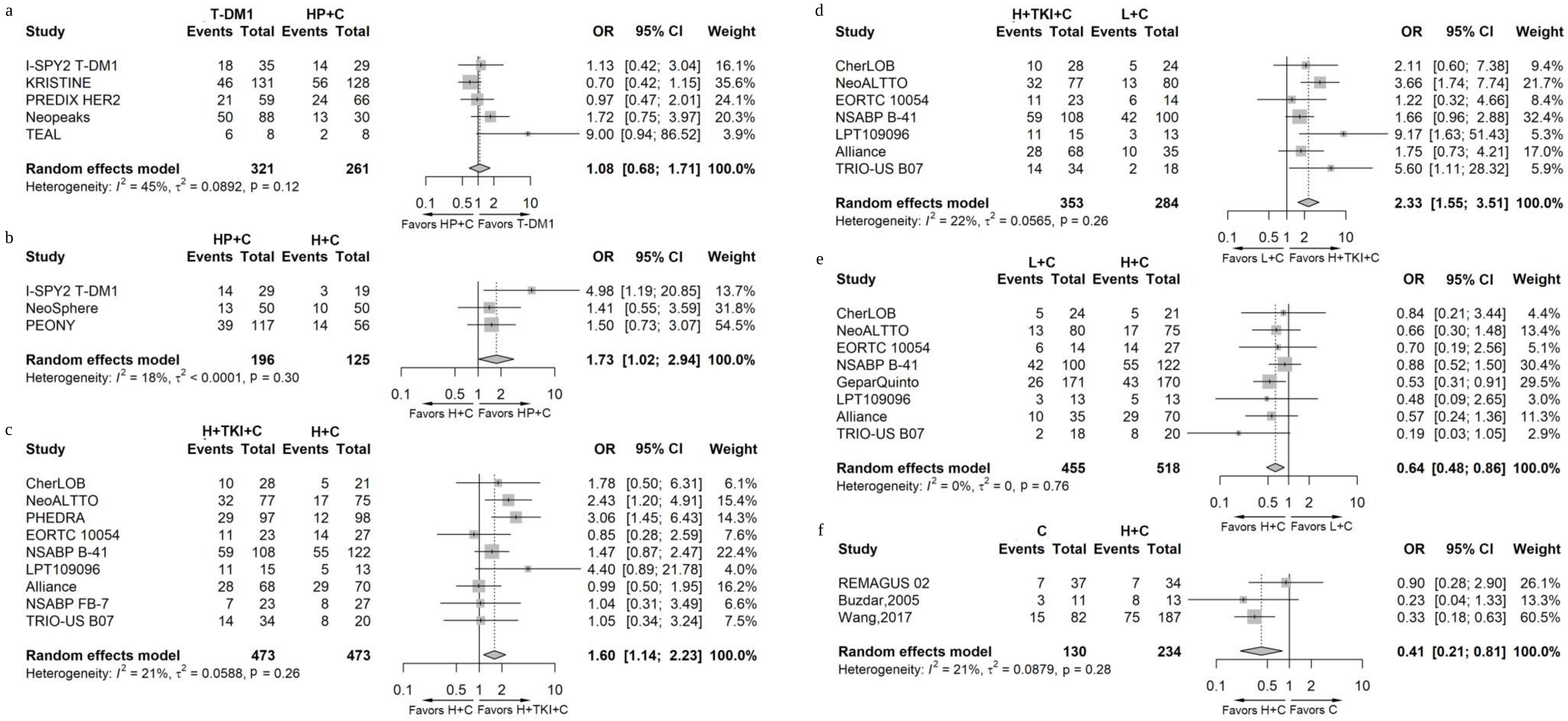

Fig. S2 Sensitivity analysis with random effects model. a: T-DM1 vs HP+C; b:HP+C vs H+C; c: H+TKI+C vs H+C; d: H+TKI+C vs L+C; e: L+C vs H+C; f: C vs H+C. T-DM1 = trastuzumab emtansine based regimens; HP+C = trastuzumab and pertuzumab with chemotherapy; H+TKI+C = trastuzumab and TKI with chemotherapy; H+C = trastuzumab with chemotherapy; L+C = lapatinib with chemotherapy; C = chemotherapy with no HER2-targeting regimen. The squares and the lines crossing the square stand for OR and its 95%CI. The diamonds stand for the estimated pooled OR and its 95%CI. Heterogeneity was tested with Cochran's Q test, and all statistical tests were two-sided.

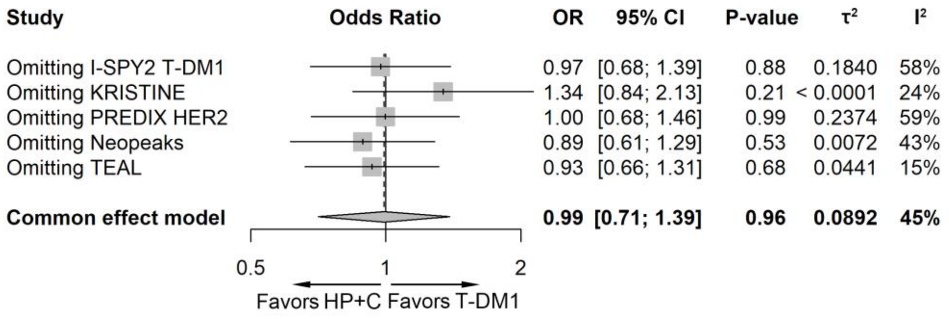

Fig. S3 Sensitivity analysis with omitting each included trial. The squares and the lines crossing the square stand for OR and its 95%CI for the result of each analysis omitting corresponding trial. The diamonds stand for the estimated pooled OR and its 95%CI with all trials included. All statistical tests were two-sided. Omitting TEAL resulted in the lowest I^2 statistic.

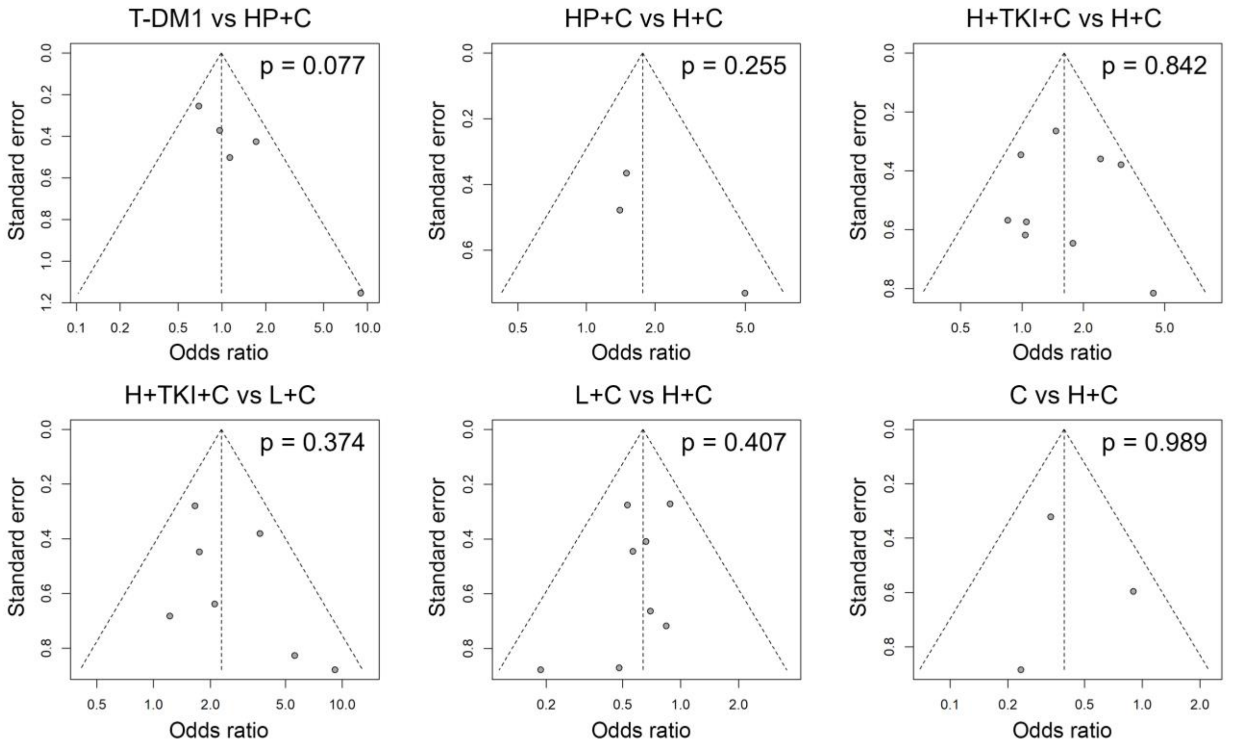

Fig. S4 Funnel plots assessing publication bias for each direct comparisons. Peter's test was conducted and p-value was calculated and presented on each plot, no significant bias was observed. T-DM1 = trastuzumab emtansine based regimens; HP+C = trastuzumab and pertuzumab with chemotherapy; H+TKI+C = trastuzumab and TKI with chemotherapy; H+C = trastuzumab with chemotherapy; L+C = lapatinib with chemotherapy; C = chemotherapy with no HER2-targeting regimen.

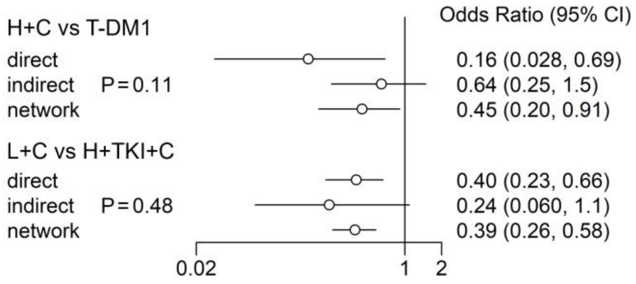

Fig. S5 Forest plot of consistency test with node-splitting model. H+C vs T-DM1 stands for the loop containing T-DM1, HP+C, and H+C; L+C vs H+TKI+C stands for the loop containing H+TKI+C, H+C, and L+C. Direct and indirect comparing result in each loop were calculated separately and the pooled OR in the network were also presented. P-values were calculated and no significant inconsistency was observed in any loop of the network. T-DM1 = trastuzumab emtansine based regimens; HP+C = trastuzumab and pertuzumab with chemotherapy; H+TKI+C = trastuzumab and TKI with chemotherapy; H+C = trastuzumab with chemotherapy; L+C = lapatinib with chemotherapy.

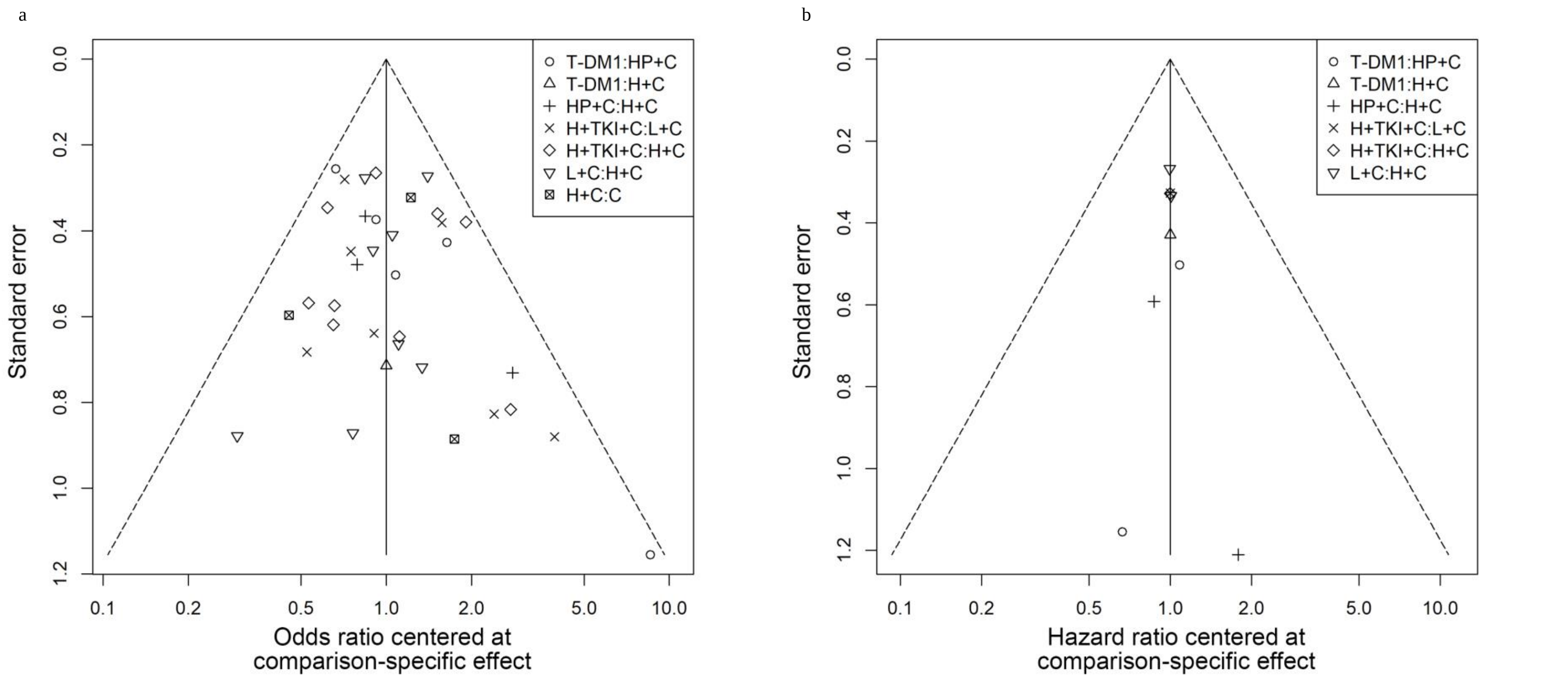

Fig. S6 Comparison-adjusted funnel plot of pCR network for a. pCR and b. EFS.

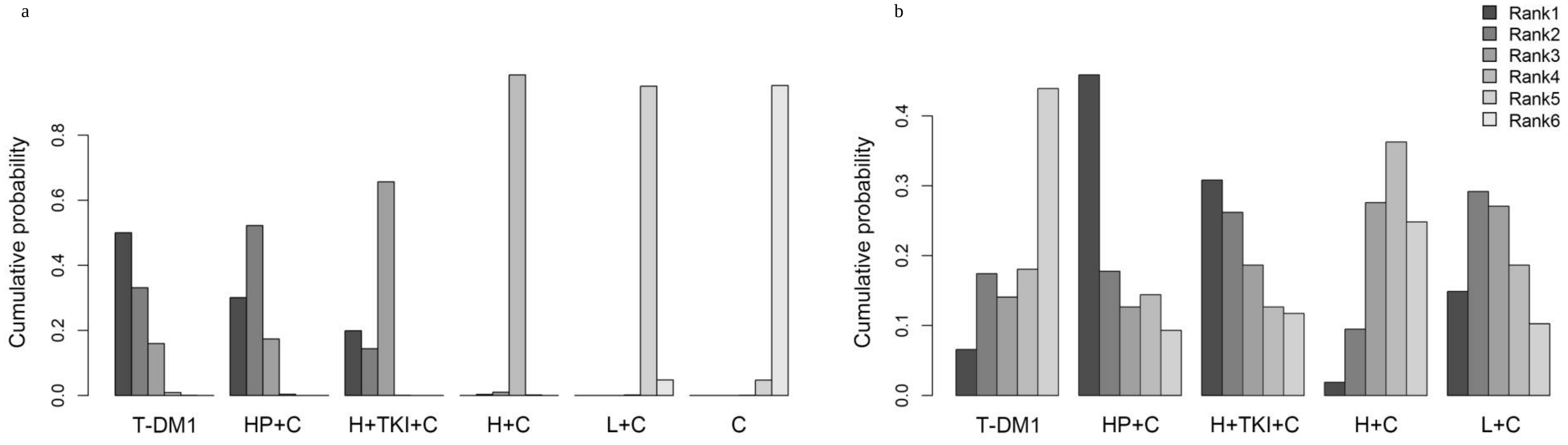

Fig. S7 Ranking probability plot for pCR and EFS comparing result. a. pCR and b. EFS. T-DM1 = trastuzumab emtansine based regimens; HP+C = trastuzumab and pertuzumab with chemotherapy; H+TKI+C = trastuzumab and TKI with chemotherapy; H+C = trastuzumab with chemotherapy; L+C = lapatinib with chemotherapy; C = chemotherapy with no HER2-targeting regimen.

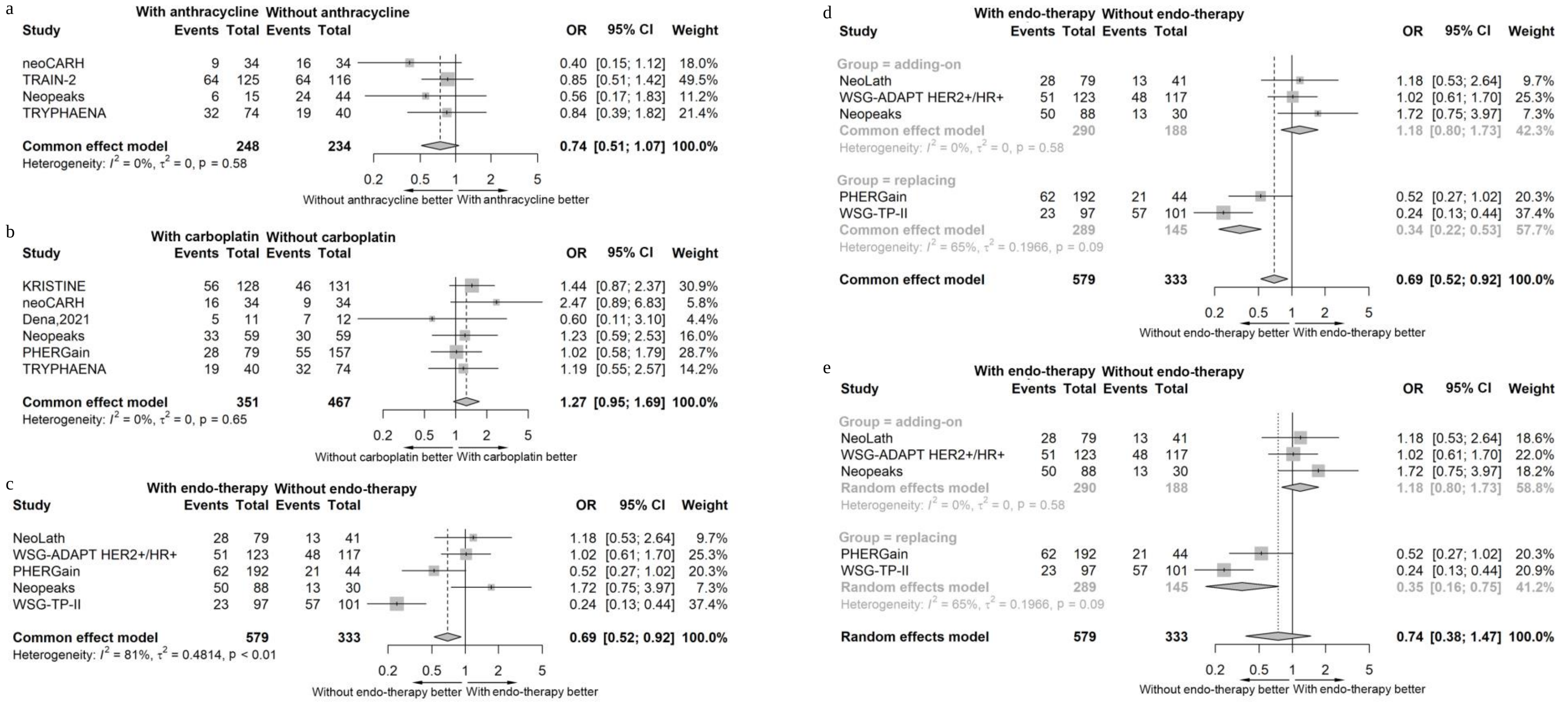

Fig. S8 Forest plots for chemotherapy and endocrine therapy analyses. a. comparing regimen with or without anthracycline; b. comparing regimen with or without carboplatin; c. comparing regimen with or without endocrine therapy; subgroup analysis differing adding endocrine therapy to standard regimen and replacing chemotherapy with endocrine therapy with d. common effect model and e. random effects model.

Types of therapy	Categories	Mechanism	Drugs
**Target therapy** (inhibits HER2 downstream signalling pathways and blocks cell cycle progression)	Antibody	Antibody-dependent cell-mediated cytotoxicity	Trastuzumab
		Inhibit HER2 dimerization and activation	Pertuzumab
	TKI agents	Competitive bind to the ATP-binding site of HER2	Lapatinib
			Tucatinib
		Covalently combine ATP-binding domain of HER2	Neratinib
			Pyrotinib
	ADC	HER2-targeting activity and targeted intracellular delivery of cytotoxic agents	T-DM1
			T-DXd
**Chemotherapy**	Alkylating agents	Inhibit the transcription of DNA to RNA	Cyclophosphamide
	Antimetabolite	Inhibit DNA synthesis	Methotrexate
			5-fluorouracil
			Gemcitabine
			Capecitabine
	Anthracycline	Intercalate with DNA and interfere with DNA metabolism and RNA production	Epirubicin
			Doxorubicin
	Taxane	Bind to microtubules and suppressing microtubule dynamics	Paclitaxel
			Docetaxel
	Platinum	Cross-link DNA strands and inhibit DNA synthesis and function	Carboplatin
**Endocrine therapy**		Inhibit estrogen action via receptor antagonism	Leuprorelin
			Tamoxifen
			Letrozole

Table S1. Drugs used for neoadjuvant treatment of breast cancer.

Section	No.	Terms
Population	#1	"breast cancer"[Title/Abstract]
	#2	"breast neoplasms"[Title/Abstract]
	#3	#1 OR #2
Intervention	#4	"neoadjuvant"[Title/Abstract] OR "preoperative"[Title/Abstract]
	#5	"Perjeta"[Title/Abstract] OR "pertuzumab"[Supplementary Concept] OR "pertuzumab"[Title/Abstract] OR "rhumab 2c4"[Title/Abstract] OR "rhumab 2c4"[Title/Abstract] OR "Omnitarg"[Title/Abstract] OR "omnitarg 2c4"[Title/Abstract] OR "pertuzumab"[Title/Abstract]
	#6	"trastuzumab"[MeSH Terms] OR "trastuzumab"[Title/Abstract] OR "herceptin"[Title/Abstract] OR "trastuzumab's"[Title/Abstract] OR "trazimera"[Title/Abstract]
	#7	"Ado Trastuzumab Emtansine"[Title/Abstract] OR "Trastuzumab Emtansine"[Title/Abstract] OR "Kadcyla"[Title/Abstract] OR "hun901 dm1"[Title/Abstract] OR "huN901DM1"[Title/Abstract] OR "trastuzumab dm1 conjugate"[Title/Abstract] OR "trastuzumab dm1"[Title/Abstract] OR "T-DM1"[Title/Abstract]
	#8	"Tykerb"[Title/Abstract] OR "Lapatinib Ditosylate"[Title/Abstract] OR "GW282974X"[Title/Abstract] OR "GW572016"[Title/Abstract] OR "gw 572016"[Title/Abstract] OR "Lapatinib"[Title/Abstract]
	#9	"HKI272"[Title/Abstract] OR "HKI-272"[Title/Abstract] OR "neratinib maleate"[Title/Abstract] OR "Nerlynx"[Title/Abstract] OR "Neratinib"[Title/Abstract]
	#10	"pyrotinib"[Title/Abstract]
	#11	"tucatinib"[Title/Abstract]
	#12	#5 OR #6 OR #7 OR #8 OR #9 OR #10 OR #11
	#13	#4 AND #12
Outcome	#14	"pathological complete response"[All Fields]
	#15	"disease-free survival"[All Fields]
	#16	"event-free survival"[All Fields]
	#17	"adverse event"[All Fields]
	#18	#14 OR #15 OR #16 OR #17
Combination	#19	#3 AND #13 AND #18

Table S2. Search strategy used in MEDLINE.

Section	No.	Terms
**Population**	#1	(breast cancer):ti,ab,kw
	#2	(breast neoplasms):ti,ab,kw
	#3	#1 OR #2
**Intervention**	#4	("neoadjuvant" OR "preoperative"):ti,ab,kw
	#5	("Perjeta" OR "pertuzumab" OR "pertuzumab" OR "rhumab 2c4" OR "rhumab 2c4" OR "Omnitarg" OR "omnitarg 2c4" OR "pertuzumab"):ti,ab,kw
	#6	("trastuzumab" OR "herceptin" OR "trastuzumab's" OR "trazimera"):ti,ab,kw
	#7	("Ado Trastuzumab Emtansine" OR "Trastuzumab Emtansine" OR "Kadcyla" OR "hun901 dm1" OR "hun901 dm1" OR "huN901DM1" OR "trastuzumab dm1 conjugate" OR "trastuzumab dm1 conjugate" OR "trastuzumab dm1" OR "trastuzumab dm1" OR "T-DM1"):ti,ab,kw
	#8	("Tykerb" OR "Lapatinib Ditosylate" OR "GW282974X" OR "GW572016" OR "gw 572016" OR "gw 572016" OR "Lapatinib"):ti,ab,kw
	#9	("HKI272" OR "HKI-272" OR "neratinib maleate" OR "Nerlynx" OR "Neratinib"):ti,ab,kw
	#10	(pyrotinib):ti,ab,kw
	#11	(tucatinib):ti,ab,kw
	#12	#5 OR #6 OR #7 OR #8 OR #9 OR #10 OR #11
	#13	#4 AND #12
**Outcome**	#14	"pathological complete response"
	#15	"disease-free survival"
	#16	"event-free survival"
	#17	"adverse event"
	#18	#14 OR #15 OR #16 OR #17
**Combination**	#19	#3 AND #13 AND #18

Table S3. Search strategy used in the Cochrane Library.

Section	No.	Terms
**Population**	#1	'breast cancer'/exp OR 'breast cancer':ab,ti
	#2	'breast neoplasms'/exp OR 'breast neoplasms':ab,ti
	#3	#1 OR #2
**Intervention**	#4	neoadjuvant:ab,ti OR preoperative:ab,ti
	#5	perjeta:ab,ti OR rhumab:ab,ti OR 'rhumab 2c4':ab,ti OR omnitarg:ab,ti OR 'omnitarg 2c4':ab,ti OR 'pertuzumab'/exp OR pertuzumab:ab,ti
	#6	'trastuzumab'/exp OR trastuzumab:ab,ti OR herceptin:ab,ti OR trazimera:ab,ti
	#7	'ado trastuzumab emtansine'/exp OR 'ado trastuzumab emtansine':ab,ti OR 'trastuzumab emtansine':ab,ti OR kadcyla:ab,ti OR 'hun901 dm1':ab,ti OR hun901dm1:ab,ti OR 'trastuzumab dm1 conjugate':ab,ti OR 'trastuzumab dm1':ab,ti OR 't dm1':ab,ti
	#8	tykerb:ab,ti OR 'lapatinib ditosylate':ab,ti OR gw282974x:ab,ti OR 'gw 572016':ab,ti OR lapatinib:ab,ti OR 'lapatinib'/exp
	#9	hki272:ab,ti OR 'hki 272':ab,ti OR 'neratinib maleate':ab,ti OR nerlynx:ab,ti OR neratinib:ab,ti OR 'neratinib'/exp
	#10	Pyrotinib:ab,ti
	#11	tucatinib:ab,ti OR 'tucatinib'/exp
	#12	#5 OR #6 OR #7 OR #8 OR #9 OR #10 OR #11
	#13	#4 AND #12
**Outcome**	#14	'disease free survival' OR 'event free survival' OR 'adverse event' OR 'pathological complete response'
**Combination**	#15	#3 AND #13 AND #14
	#16	#15 AND ([article]/lim OR [article in press]/lim OR [preprint]/lim)

Table S4. Search strategy used in Embase.

Section	No.	Terms
Population	#1	AB=(breast cancer) OR TI=(breast cancer)
	#2	AB=(breast neoplasms) OR TI=(breast neoplasms)
	#3	#1 OR #2
Intervention	#4	AB=(neoadjuvant) OR AB=(preoperative)
	#5	AB=("Perjeta" OR "pertuzumab" OR "pertuzumab" OR "rhumab 2c4" OR "rhumab 2c4" OR "Omnitarg" OR "omnitarg 2c4" OR "pertuzumab”)
	#6	AB=("trastuzumab" OR "herceptin" OR "trastuzumab's" OR "trazimera”)
	#7	AB=("Ado Trastuzumab Emtansine" OR "Trastuzumab Emtansine" OR "Kadcyla" OR "hun901 dm1" OR "hun901 dm1" OR "huN901DM1" OR "trastuzumab dm1 conjugate" OR "trastuzumab dm1 conjugate" OR "trastuzumab dm1" OR "trastuzumab dm1" OR "T-DM1”)
	#8	AB=("Tykerb" OR "Lapatinib Ditosylate" OR "GW282974X" OR "GW572016" OR "gw 572016" OR "gw 572016" OR "Lapatinib”)
	#9	AB=("HKI272" OR "HKI-272" OR "neratinib maleate" OR "Nerlynx" OR "Neratinib”)
	#10	AB=pyrotinib
	#11	AB=tucatinib
	#12	#5 OR #6 OR #7 OR #8 OR #9 OR #10 OR #11
	#13	#4 AND #12
Outcome	#14	TS=("pathological complete response")
	#15	TS=("disease free survival")
	#16	TS=("event free survival")
	#17	TS=("adverse event")
	#18	#14 OR #15 OR #16 OR #17
Combination	#19	#3 AND #13 AND #18

Table S5. Search strategy used in Web of Science.

Study	Randomization Process	Deviations from Intended Interventions	Missing Outcome Data	Measurement of the Outcome	Selection of the Reported Result	Overall
**CherLOB**	Low	Some concerns	Low	Low	Low	Some concerns
**I-SPY2 T-DM1**	Low	Low	Low	Low	Low	Low
**KRISTINE**	Low	Low	Low	Low	Low	Low
**NeoALTTO**	Low	Low	Low	Low	Low	Low
**NeoSphere**	Low	Some concerns	Low	Low	Low	Some concerns
**PHEDRA**	Low	Low	Low	Low	Low	Low
**REMAGUS 02**	Low	Low	Low	Low	Low	Low
**WSG-ADAPT HER2+/HR+**	Low	Low	Low	Low	Low	Low
**EORTC 10054**	Low	Some concerns	Low	Low	Low	Some concerns
**PREDIX HER2**	Low	Low	Low	Low	Low	Low
**NSABP B-41**	Low	Low	Low	Low	Low	Low
**Neopeaks**	Low	Low	Low	Low	Low	Low
**Buzdar, 2005**	Low	Some concerns	Low	Low	Low	Some concerns
**PEONY**	Low	Low	Low	Low	Low	Low
**GeparQuinto**	Low	Low	Low	Low	Low	Low
**LPT109096**	Low	Low	Low	Low	Low	Low
**NSABP FB-7**	Low	Low	Low	Low	Low	Low
**TRIO-US B07**	Low	Some concerns	Low	Low	Low	Some concerns
**TEAL**	Low	Low	Low	Low	Some concerns	Some concerns
**CALGB 40601**	Low	Low	Low	Low	Low	Low
**Wang, 2017**	High	Low	Low	Low	Low	High
**NeoLaTH**	Low	Low	Low	Low	Low	Low
**WSG-TP-II**	Low	Low	Low	Low	Low	Low
**Dena, 2021**	Low	Low	Low	Low	Low	Low
**PHERGain**	Low	Low	Low	Low	Low	Low
**neoCARH**	Low	Low	Low	Low	Low	Low
**TRAIN-2**	Low	Low	Low	Low	Low	Low
**TRYPHAENA**	Low	Low	Low	Low	Low	Low

Table S6. Detailed risk of bias assessment results of each trial.

Comparison	Comparing results		Heterogeneity test	
	Common effect model	Random effects model	I^2 statistic	p-value
**T-DM1 vs HP+C**	0.99 (0.71-1.39)	1.08 (0.68-1.71)	45%	0.12
**HP+C vs H+C**	1.77 (1.05-2.98)	1.73 (1.02-2.94)	18%	0.30
**H+TKI+C vs H+C**	1.61 (1.22-2.11)	1.60 (1.14-2.23)	21%	0.26
**H+TKI+C vs L+C**	2.29 (1.63-3.21)	2.33 (1.55-3.51)	22%	0.26
**L+C vs H+C**	0.64 (0.48-0.85)	0.64 (0.48-0.86)	0%	0.76
**C vs H+C**	0.39 (0.23-0.66)	0.41 (0.21-0.81)	21%	0.28

Table S7. Summarized results of sensitivity analyses of direct comparisons. T-DM1 = trastuzumab emtansine based regimens; HP+C = trastuzumab and pertuzumab with chemotherapy; H+TKI+C = trastuzumab and TKI with chemotherapy; H+C = trastuzumab with chemotherapy; L+C = lapatinib with chemotherapy; C = chemotherapy with no HER2-targeting regimen.

Regimens	T-DM1				
T-DM1		HP+C			
HP+C	1.04 (0.74, 1.46)		H+TKI+C		
H+TKI+C	1.22 (0.65, 2.38)	1.18 (0.67, 2.14)		H+C	
H+C	**1.95 (1.10, 3.63)**	**1.87 (1.14, 3.22)**	**1.60 (1.22, 2.11)**		L+C
L+C	**3.03 (1.58, 5.94)**	**2.90 (1.65, 5.33)**	**2.48 (1.82, 3.40)**	**1.55 (1.16, 2.07)**	
C	**3.49 (1.12, 11.19)**	**3.37 (1.13, 10.35)**	**2.85 (1.05, 7.98)**	1.79 (0.68, 4.87)	1.15 (0.42, 3.25)

Table S8. Odds ratios with 95% confidence intervals comparing pCR in sensitivity analysis removing trials with high risk of overall bias. Results were compared from top to left with significant difference in bold font. T-DM1 = trastuzumab emtansine based regimens; HP+C = trastuzumab and pertuzumab with chemotherapy; H+TKI+C = trastuzumab and TKI with chemotherapy; H+C = trastuzumab with chemotherapy; L+C = lapatinib with chemotherapy; C = chemotherapy with no HER2-targeting regimen.

**Regimens**	**T-DM1**				
**T-DM1**		**HP+C**			
**HP+C**	1.04 (0.74, 1.46)		**H+TKI+C**		
**H+TKI+C**	1.35 (0.69, 2.66)	1.30 (0.71, 2.38)		**H+C**	
**H+C**	**1.97 (1.09, 3.58)**	**1.90 (1.13, 3.19)**	**1.47 (1.07, 1.98)**		**L+C**
**L+C**	**3.18 (1.63, 6.18)**	**3.05 (1.68, 5.54)**	**2.35 (1.70, 3.27)**	**1.60 (1.21, 2.17)**	
**C**	**5.14 (2.30, 11.53)**	**4.92 (2.32, 10.44)**	**3.78 (2.06, 7.08)**	**2.58 (1.54, 4.47)**	1.61 (0.88, 2.99)

Table S9. Odds ratios with 95% confidence intervals comparing pCR in sensitivity analysis removing trials using other TKIs instead of lapatinib. Results were compared from top to left with significant difference in bold font. T-DM1 = trastuzumab emtansine based regimens; HP+C = trastuzumab and pertuzumab with chemotherapy; H+TKI+C = trastuzumab and TKI with chemotherapy; H+C = trastuzumab with chemotherapy; L+C = lapatinib with chemotherapy; C = chemotherapy with no HER2-targeting regimen.

Regimens	T-DM1				
T-DM1		HP+C			
HP+C	1.03 (0.74, 1.45)		H+TKI+C		
H+TKI+C	1.44 (0.68, 3.11)	1.39 (0.70, 2.89)		H+C	
H+C	**2.21 (1.12, 4.46)**	**2.13 (1.14, 4.15)**	**1.52 (1.13, 2.06)**		L+C
L+C	**3.06 (1.39, 6.80)**	**2.95 (1.41, 6.29)**	**2.11 (1.44, 3.08)**	1.38 (0.95, 2.02)	
C	**5.71 (2.45, 13.82)**	**5.54 (2.45, 12.86)**	**3.95 (2.18, 7.28)**	**2.58 (1.54, 4.44)**	1.87 (0.97, 3.61)

Table S10. Odds ratios with 95% confidence intervals comparing pCR in sensitivity analysis removing trials reporting pCR outcomes in different definitions. Results were compared from top to left with significant difference in bold font. T-DM1 = trastuzumab emtansine based regimens; HP+C = trastuzumab and pertuzumab with chemotherapy; H+TKI+C = trastuzumab and TKI with chemotherapy; H+C = trastuzumab with chemotherapy; L+C = lapatinib with chemotherapy; C = chemotherapy with no HER2-targeting regimen.
